# New data on the rare Afrotropical scarab beetles *Orphnus drumonti* Frolov and *Delopleurus naviauxi* Frolov et Cambefort (Coleoptera, Scarabaeidae, Orphninae and Scarabaeinae)

**DOI:** 10.3897/BDJ.3.e5444

**Published:** 2015-08-10

**Authors:** Andrey V. Frolov, Lilia A. Akhmetova

**Affiliations:** ‡ Zoological Institute RAS, St. Petersburg, Russia

**Keywords:** Scarab beetles, scarabaeines, orphnines, new locality record, description, Africa, Afrotropical Region, Democratic Republic of Congo, Tanzania

## Abstract

**Background:**

The scarab beetle genera *Orphnus* Macleay and *Delopleurus* Boheman are most speciose in the Afrotropical region. However, a number of species are only known from type specimens, sometimes only from one sex.

**New information:**

New locality records of *Orphnus drumonti* Frolov (Orphninae) and *Delopleurus naviauxi* Frolov et Cambefort (Scarabaeinae) are given. The previously unknown female of the former and male of the latter species are described.

## Introduction

*Orphnus* Macleay, 1819, is the most speciose genus of the scarab beetle subfamily Orphninae comprising currently about 100 nominal species (Paulian 1948, Petrovitz 1971). Most of the *Orphnus* species have a more or less developed head and pronotal armature. One of the two species with a strongly bifurcated clypeal horn, *O. drumonti* Frolov, 2009, has so far been known from a single type specimen (Frolov 2009).

*Delopleurus* Erichson, 1847, comprises 10 superficially similar, small-sized species distributed in the Afrotropical region and Southern Asia (Frolov 2014, Král 2014). The genus is commonly classed as dung beetles, however, there is no evidence of its association with dung but rather with higher fungi. Most of the *Delopleurus* species occur in the Afrotropical Region and a few of them are known only from the type material. Of them *Delopleurus naviauxi* Frolov et Cambefort in Frolov, 2014, was described from two females and no more material was available until recently.

Examination of the collections of the museums listed below revealed additional material which provided new locality records of the two species and allowed descriptions of the sexes lacked in the type series.

## Materials and methods

Examined material is housed in the National Museum of Natural History “Naturalis”, Leiden (RMNH) and Zoological Museum of University of Copenhagen, Copenhagen (ZMUC).

Preparation of genitalia follows the common technique used in entomological research. Partially focused serial images were combined in Helicon Focus software (Helicon Soft Ltd.) to produce completely focused images. The photographs were not altered except for levels and tone correction in Adobe Photoshop. The distribution map was generated with ArcGIS software. Co-ordinates of the localities were taken from the specimens labels, if available, or from the NGA GEOnet Names Server (GNS, geonames.nga.mil/gns/html/). Ecoregion names follow Olson et al. (2001).

## Taxon treatments

### Orphnus drumonti

Frolov 2009

#### Materials

- **Type status:** Other material. **Occurrence:** individualCount: 2; sex: 1 male, 1 female; lifeStage: adult; **Taxon:** scientificName: *Orphnus drumonti* Frolov, 2009; namePublishedIn: Frolov, A.V. 2009. Orphnus drumonti sp. n. (Coleoptera, Scarabaeidae) iz Demokraticheskoj Respubliki Kongo. Zoologicheskii Zhurnal, 88 (9): 1137‑1138; kingdom: Animalia; class: Insecta; order: Coleoptera; family: Scarabaeidae; genus: Orphnus; specificEpithet: drumonti; taxonRank: species; taxonomicStatus: valid; **Location:** continent: Africa; county: Democratic Republic of Congo; locality: Kimwenza; decimalLatitude: -4.4592; decimalLongitude: 15.2889; **Identification:** identifiedBy: Andrey Frolov; **Event:** year: 1956; **Record Level:** collectionID: urn:lsid:biocol.org:col:34992; institutionCode: RMNH

#### Description

##### Description of female

**Figure 1.**
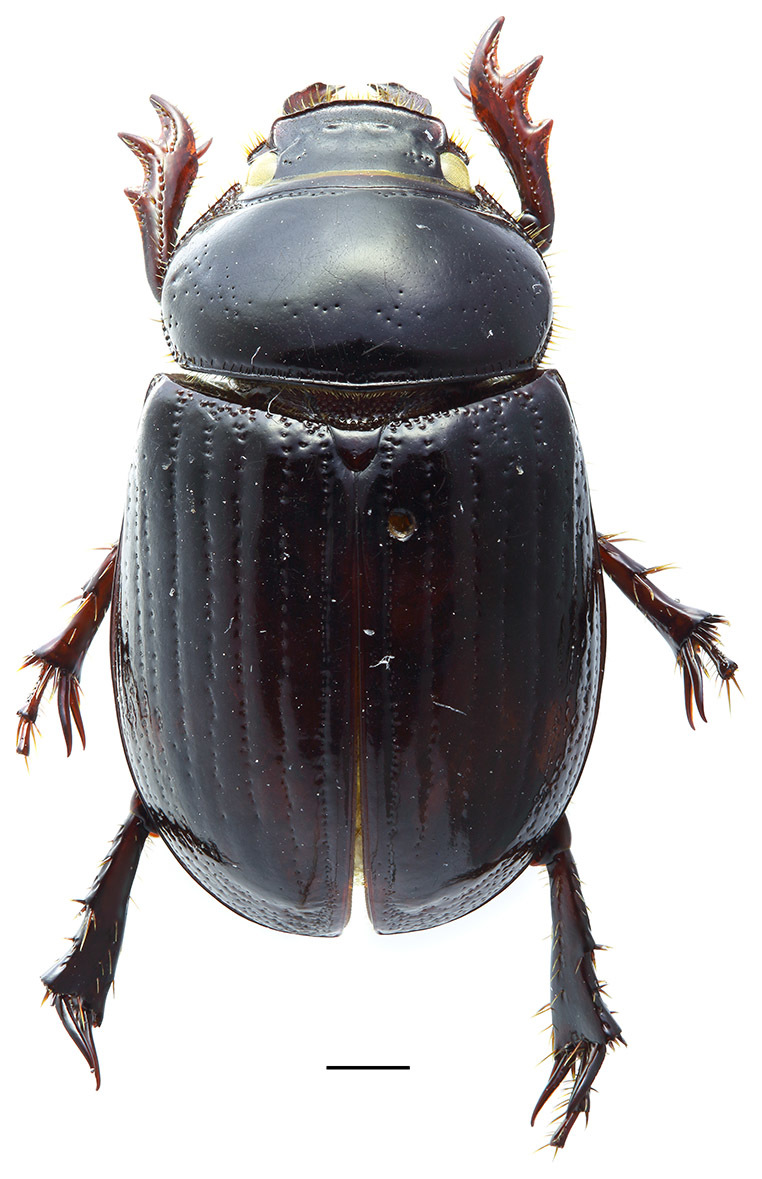
**Orphnus drumonti**, female, habitus. Scale 1 mm.

Medium-sized beetle (length 11.0 mm, width 6.3 mm) with strongly shiny, blackish-brown body (Fig. 1).

**Figure 2.**
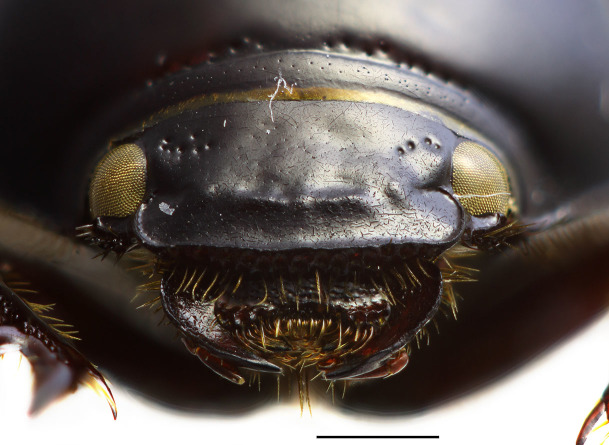
*Orphnus drumonti*, female, head. Scale 1 mm.

Clypeus widely rounded anteriorly, obtusely rounded laterally. Genae very small, not protruding past eyes. Anterior part of clypeus with low transverse ridge occupying about 1/3 width of clypeus. Dorsal side of head smooth with a few coarse punctures near eyes (Fig. 2).

Pronotum trapezoidal, with rounded lateral margins, about 1.8 times wider than long, convex, without excavations or ridges. Dorsal surface of pronotum with minute, feebly visible punctures throughout and with sparse, coarser punctures mostly laterally on disc. Lateral sides somewhat crenulate in dorsal view. Anterior margin with wide border and row of coarse punctures on disc along border. Basal margin with fine border and row of longitudinally elongated punctures along border.

Elytra strongly convex, with rows of large punctures along striae. Elytral intervals convex, with fine sparse punctation.

Scutellum rounded apically, smooth, about 1/11 the length of elytra.

Wings fully developed.

Anterior tibiae with 3 long outer teeth and a robust apical spur. Lateral margin basad of outer teeth not crenulate. Ventral surface of anterior tibiae smooth with two rows of setae along sides and a few very long setae in the middle. Middle and posterior legs similar in shape; posterior femora and tibiae about 1/8 longer than the middle ones. Middle and posterior femora almost impunctate, with 2 apical spurs; inner margin slightly concave with one transverse keel.

Abdominal sternites irregularly punctate, pubescent with sparse long setae. Visible sternite 6 as long as sternites 4–5 together in middle.

Pygidium semi-hidden under elytra, with sparse punctures and sparse long setae.

##### Sexual dimorphism

**Figure 3.**
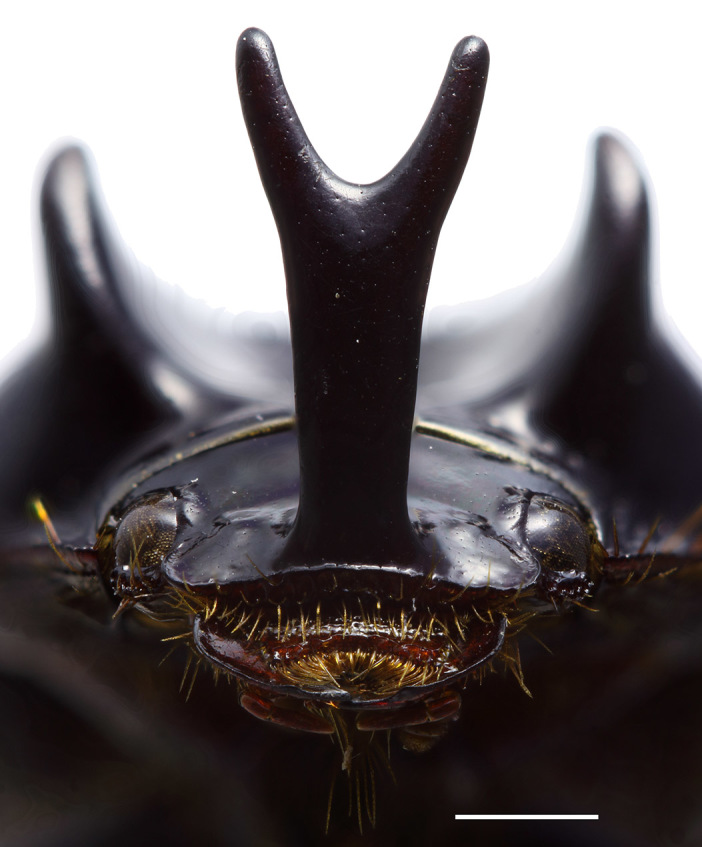
*Orphnus drumonti*, male, head. Scale 1 mm.

Female differs from male in having distinct protibial spur, relatively wider elytra, low transverse clypeal carina, and convex pronotum without excavations or ridges. Male have no protibial spur, narrower elytra (about as wide as pronotum), deep pronotal excavation with long ridges aside, and long, apically bifurcated clypeal horn (Fig. 3).

#### Distribution

**Figure 4.**
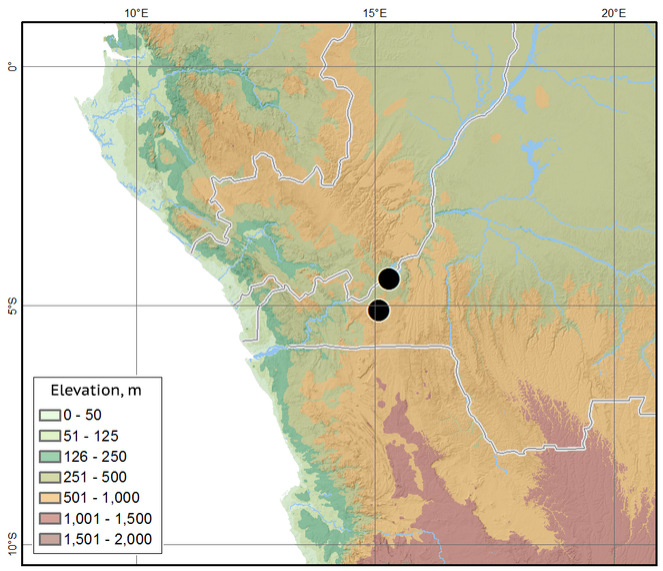
*Orphnus drumonti*, locality map.

The species was described from Kisantu in the western part of Democratic Republic of Congo. The new locality is some 70 km north, in the same Western Congolian forest-savanna mosaic ecoregion (Fig. 4).

### Delopleurus naviauxi

Frolov et Cambefort in Frolov, 2014

#### Materials

- **Type status:** Other material. **Occurrence:** recordedBy: Sofie Tind Nielsen; individualCount: 3; sex: 2 males, 1 female; **Taxon:** scientificName: Delopleurus naviauxi Frolov et Cambefort in Frolov, 2014; namePublishedIn: Frolov, A. V. (2014) Revision of the genus Delopleurus Erichson (Coleoptera: Scarabaeidae: Scarabaeinae) with description of new species from Africa. Journal of Natural History, 49, 129-154.; kingdom: Animalia; class: Insecta; order: Coleoptera; family: Scarabaeidae; genus: Delopleurus; taxonomicStatus: valid; **Location:** continent: Africa; country: Tanzania; county: Morogoro Region; locality: Majawanga; verbatimLocality: Majawanga village; verbatimElevation: 1400 m; decimalLatitude: -6.083488; decimalLongitude: 36.9; **Identification:** identifiedBy: Andrey Frolov; **Event:** verbatimEventDate: 14-18.III.2004; **Record Level:** institutionCode: ZMUC

#### Description

##### Description of male

**Figure 5.**
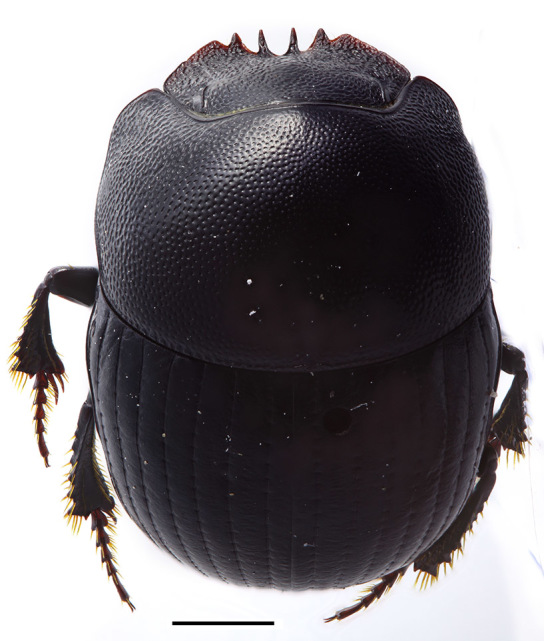
*Delopleurus naviauxi*, male, habitus. Scale 1 mm.

Body (Fig. 5) strongly convex, black, glabrous, length 4.7–5.5 mm.

Clypeus quadridentate. All four clypeal teeth acute, two medial ones relatively slender. Head without carinae on disc, small carinae present near inner margin of eyes. Genae right-angled, indistinctly separated from clypeus. Frontoclypeal and genal suturae indistinct. Eyes small, their dorsal parts slit-shaped, ventral parts sub-rectangular. Clypeus rugose in anterior part and laterally, frons densely punctate with elongate punctures.

Pronotum trapezoidal, about 2 times wider than long. Anterior and lateral margins bordered, base without border. Pronotum relatively densely punctate on disc (punctures separated by 1-2 puncture diameters), punctation becoming denser laterally.

Elytra trapezoidal, as wide as long, shiny. Striae distinct, with punctures larger than striae. Elytral intervals slightly convex on disc, with minute punctation.

**Figure 6.**
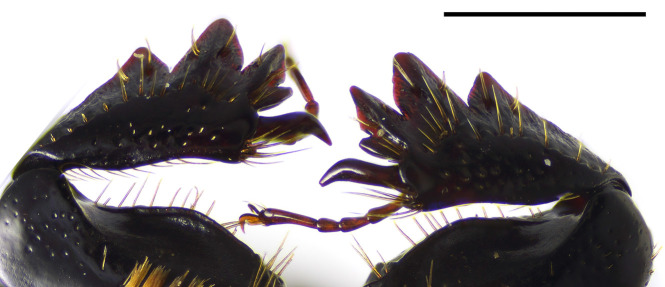
*Delopleurus naviauxi*, male, protibiae. Scale 1 mm.

Anterior tibiae with 3 outer teeth and a small acute tooth between 1st outer tooth and apical spur (Fig. 6).

**Figure 7.**
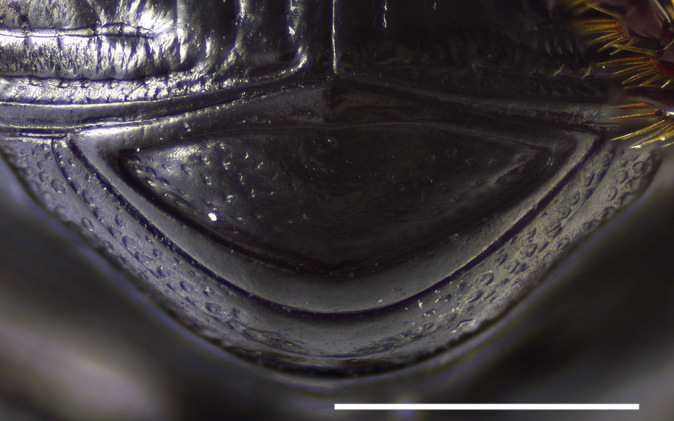
*Delopleurus naviauxi*, male, pygidium. Scale 1 mm.

Pygidium with relatively slender borders and convex disc (Fig. 7). Basal border slender and almost parallel-sided except in the middle. Apical border about 2 times thicker in the middle than the basal border, becoming more slender laterally. Disc glabrous, punctate with punctures separated by 1–2 puncture diameters.

**Figure 8.**
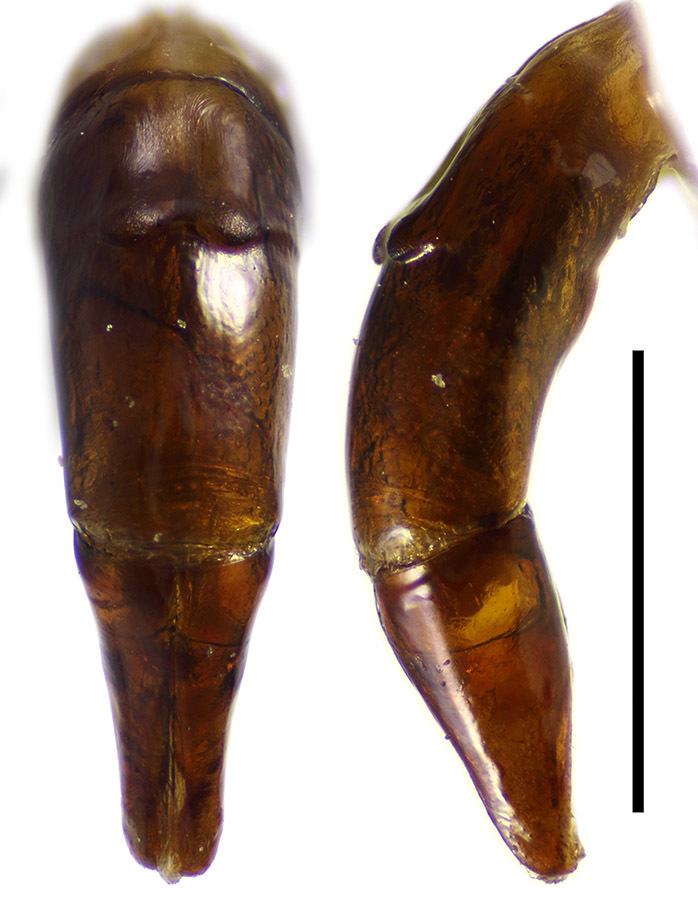
*Delopleurus naviauxi*, male, aedeagus in dorsal and lateral view. Scale 1 mm.

Aedeagus of typical scarabaeine shape (Fig. 8). Phallobase with two symmetrical tubercles dorsally. Parameres symmetrical, without setae apically, rounded in lateral view.

##### Sexual dimorphism

Male differs from female in having pygidium with relatively large disc without visible setae as opposed to being with a deep transversal slit-shaped fossa with yellowish setae in female.

#### Distribution

**Figure 9.**
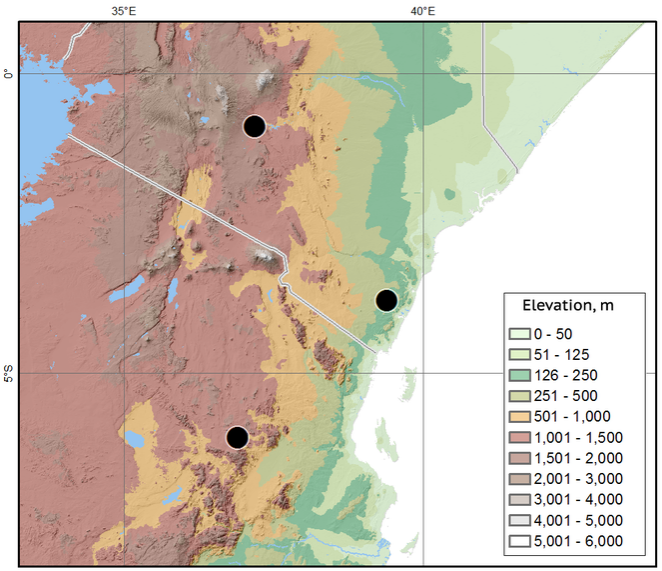
*Delopleurus naviauxi*, locality map.

The species was described from two localities in the Northern Acacia-Commiphora bushlands and thickets ecoregion in Kenya (Frolov 2014). The new locality is in the Southern Acacia-Commiphora bushlands and thickets ecoregion in Tanzania (Fig. 9).

## Supplementary Material

XML Treatment for Orphnus drumonti

XML Treatment for Delopleurus naviauxi
